# Experimental and DEM-Based Numerical Studies on the Shearing Characteristics of Talus-like Rock Mass

**DOI:** 10.3390/ma15186372

**Published:** 2022-09-14

**Authors:** Xiaochang Li, Zixin Zhang, Yinlian Yi, Shuaifeng Wang

**Affiliations:** 1 Department of Geotechnical Engineering, College of Civil Engineering, Tongji University, Shanghai 200092, China; 2 Key Laboratory of Geotechnical and Underground Engineering, Ministry of Education, Tongji University, Shanghai 200092, China

**Keywords:** talus-like rock mass, shearing test, DEM simulation, shearing characteristics, dilatancy, force chain, coordination number, bond-break

## Abstract

The talus-like rock mass is a special kind of geomaterial widely distributed in southwestern China, which has induced serious engineering disasters for tunneling engineering. However, the mechanical behavior of the talus-like rock mass remains unclear as the previous studies mainly focused on similar geomaterials such as the soil–rock mixtures. In this paper, we have carried out both experimental and discrete element method (DEM)-based numerical analyses to investigate the shearing characteristics of the talus-like rock mass collected from a real project site. Large-scale direct shear tests reveal that the strength parameters increase with the block content, which is different from the traditional soil–rock mixture. A dependence has been discovered in that the specimen dilation becomes more obvious under lower normal stress and larger block content. It is also observed that higher normal stress is beneficial for crushing blocks. The force chains obtained in the DEM simulations show that distinct internal structures are generated in the rock samples with different block contents. The distribution of coordination number establishes the dependence of fabric stability on block content during shearing. Bond-break evolution reveals the tendencies of crushed particles were consistent with those of experimental tests. The findings provide a more in-depth understanding about the mechanical behavior of the talus-like rock mass, which helps to uncover the cause of the collapse of the real tunnel project.

## 1. Introduction

For most regions in southwestern China, the geological conditions are more complex and the rock mass is extremely fragmentized because of the high altitudes, diverse topography and landforms, intricate geological tectonic belts and abundant groundwater resources. These special geological conditions gave birth to some special kinds of rock masses or geologic structures, such as the layered phyllite strata [1], soil–rock mixture [2] and some other weak rock masses. In addition, another special kind of rock mass called the talus-like rock mass [3], comprised of a mass of rock blocks with diverse sizes and weak intercalated layers with fine grains or soils with different fractions, is also widely distributed in southwestern China. Unlike the traditional weak rock mass or soil–rock mixture, the talus-like rock mass is usually encountered in the elurium, colluvium, and diluvial layers of Quaternary System. The talus-like rock mass can be characterized as a kind of very loose and unstable geomaterial due to the random distribution of weak interlayers of fine grains with various sizes and fractions, e.g., composed of slate and weak interlayer filled with mudstone with a highly loose and fragile structure of low strength, which is different from the traditional soil–rock mixture (often called talus formations). Therefore, we call this special type of composite mass a talus-like rock mass, for which a detailed description can be found in our previous study [3]. The Tayi Tunnel of the Jian-Ge-Yuan Highway Project in Yunnan Province of China mainly passed through the talus-like rock mass and suffered severe engineering hazards including large deformation of the primary lining and the collapse of the excavation face [3]. Therefore, it is important to investigate the mechanical properties of the talus-like rock mass, which will be beneficial to the design of tunnel supports and disaster mitigation during tunnel construction.

Generally, in order to explore the mechanical properties and obtain the mechanical parameters of geomaterials, the in-situ survey and laboratory tests were always utilized [4,5]. Recent studies about geomaterials usually pay attention to the traditional soil–rock mixture. The weight proportion, size distribution of rock fragments and water content obviously influenced the deformation and mechanical characteristics of the soil–rock mixture according to the in-situ tests [2]. In-situ shear tests regarding the complex bimrock indicated that there was a good linear positive correlation between the volumetric block content (VBC) and the bimrock friction angle, while the cohesion seemed to be strongly affected by a critical VBC threshold [6]. The possible correlations between the scarce direct information from the in-situ shear test data and the large indirect information from the synthetic image parameters were presented based on geostatistics [7]. Xu et al. [8] studied the shear strength of soil–rock mixture by large scale in-situ direct shear tests, showing that the rock block size proportion controlled the deformation and fracture mechanism of the mixture. Similar phenomena were also presented by large-scale compaction and in-situ direct shear tests [9]. Although the in-situ tests may give relative accurate results of the undisturbed geomaterial, they are always time-consuming and highly dependent on the site condition. Moreover, the accuracy of the determined block content during the in-situ tests is debatable. Thus, the laboratory tests are more frequently used to study the geomaterial because of the diversity of test methods given that the testing specimen size is larger than representative elementary volume. Generally, the direct shear test has been widely used to investigate the mixture of geomaterials. By using the large direct shear test [10], the effects of particle size on the shear behavior of coarse-grained soils with geogrid reinforcements were evaluated. The effects of gravel content on the shearing characteristics of gravelly soils were investigated by a series of shear tests on drained samples [11]. A positive relationship between the shear strength of soil–rock mixtures and the maximum particle diameter was reported [12]. The specific impacts of scale effect on the shear behaviors of soil–rock mixture were explored by large-scale laboratory direct shear tests and gave a suitable scaling method was proposed [13]. By using the laboratory meso-scale direct shear tests, Avşar [14] analyzed the shear strength parameters and the deformation behavior of a highly welded bimrock. The shear characteristics of soil–rock mixture and bedrock interphases were studied and explained based on the large direct shear test [15]. The uniaxial and triaxial tests were also employed to study the welded or unwelded mixtures. Kalender et al. [16] performed laboratory tests on the unwelded bimrocks and bimsoils. Accordingly, they developed a preliminary bim strength criterion for predicting the overall strength of bimrocks. The uniaxial compression tests on bimrocks with high rock block proportion demonstrated the relationships between the mechanical parameters and rock block proportion [17]. Both uniaxial and direct shear tests were used to validate the prediction accuracy of a new proposed empirical approach [18]. The large-scale triaxial tests on the purified soil–rock mixtures combined with some other research results systematically analyzed the influence of different factors on the mechanical behavior of soil–rock mixture [19]. Large-scale triaxial apparatus were used to study the effects of rock block content and confining pressure on the dynamic characteristics of soil–rock mixture [20]. By a series of triaxial tests, the influence of rock block content, sample scale, and “oversize rock block” processing methods on the mechanical behaviors of soil–rock mixtures were studied [21]. Considering gravel content and shape distribution, the gravel effects on shear behavior of soil–rock mixture were investigated by triaxial tests [22]. Rybak et al. [23] predicted the rock mass behavior during salt mining depending on the strength of the pillars and the strength characteristics of the stowing. Most of the experimental studies on the soil–rock mixtures included the rock content or volumetric block proportion (VBP), which is one of the most important factors that influence the mechanical behaviors of soil–rock mixture. However, in the above studies, they only focused on a limited range of values of VBP, i.e., 20%–80% [16,19,21,22]. The mechanical properties of pure fine grains or rock component are rarely studied [11], neither is the interaction between them, which may be of great importance in the analysis of the talus-like rock mass because of the random and widespread distributions of fine grains and rock blocks in this special rock mass.

The experimental method enables us to investigate the overall mechanical behavior of geomaterials. However, it is unable to capture the microscopic characteristics inside the geomaterials. Therefore, the DEM (discrete element method) has been developed to be another useful tool to study the particle-formed geomaterials such as soil–rock mixture [24,25,26]. The numerical direct shear tests were conducted for outwash deposits with random structure and composition using DEM [27]. The microanalysis on the shear deformation and strength of the interphase between the soil–rock mixture and the benched bedrock were carried out using PFC2D [28]. A discrete element model of soil–rock mixture was established and the influence of block content on the meso-structure evolution were analyzed [29]. The DEM method simulated the evolution characteristics of the shear band in soil–rock mixture based on particle rotation analysis [30]. A stochastic approach for 3D DEM modelling of the soil–rock mixture sample accounting for the morphological features and internal fractures of blocks was proposed [31]. Afterwards, the influence of block form on the shear behavior of soft soil–rock mixture was analyzed according to the proposed approach [32]. A series of DEM numerical analyses were performed to investigate the mechanical behaviors and failure mechanism of soil–rock mixture with different rock block proportions [33]. Accordingly, the DEM method is able to reproduce the laboratory test results and more insightfully investigate the microscopic features of the talus-like rock mass. Although the mechanical characteristics of the traditional soil–rock mixture have been thoroughly studied, the mechanical properties of this special type of talus-like rock mass are still unclear, which brings uncertainty and hidden danger to the supporting structure design of tunnels in such a type of rock mass. This motivates the development of our current work.

In this paper, we adopted the laboratory large-scale direct shear test and DEM-based numerical simulation to investigate the shearing characteristics of the talus-like rock mass. Firstly, three types of samples from the talus-like rock mass with different block contents, namely the mixture, fine grain and rocky block samples, were tested by large-scale shear tests. The shearing characteristics and mechanical parameters were analyzed and compared. Afterwards, the DEM models were established corresponding to the shear tests and the mechanical behaviors of the talus-like rock mass were analyzed. The results of the laboratory tests and DEM models were also compared and discussed, which gave deeper understanding about the talus-like rock mass.

## 2. Large-Scale Direct Shear Test

### 2.1. Testing Apparatus

**Figure 1:**
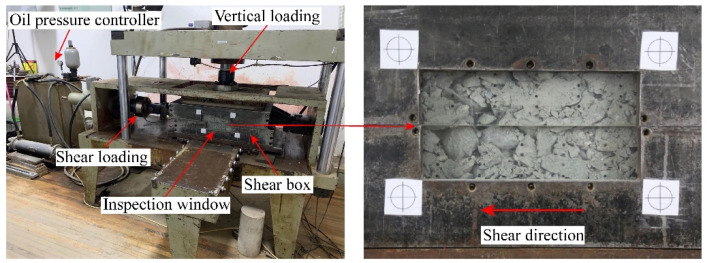
Large-scale interface shearing apparatus.

**Figure 2:**
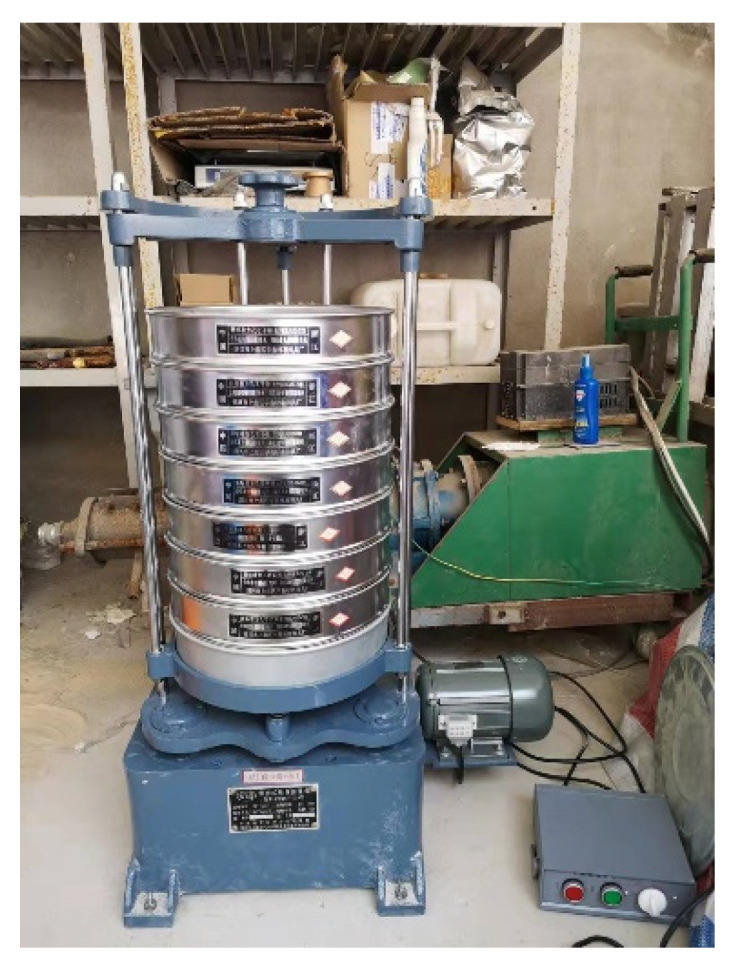
Standard vibrating screen machine.

The large-scale direct shear tests in this paper were performed by the large-scale interface shearing apparatus in Tongji University as shown in Figure 1. The apparatus consists of a large shear box (length *L* = 600 mm, width *W* = 400 mm and height *H* = 200 mm), vertical and horizontal loading units, a steel frame, an oil pressure controller, and a control/data acquisition system. The vertical rectangular loading plate above the shear box provides the vertical pressure by moving down through a high-precision oil cylinder with a capacity of 100 kN. During the shearing process, a horizontal traction load is applied to the left side of the lower shear box while keeping the upper shear box fixed. All the load and displacement data are monitored through the load cell installed at the end of the loading system. A standard vibrating screen machine with standard square-hole test sieve (Figure 2) was utilized to sieve the shear specimen to obtain the grading curve before and after shearing test.

### 2.2. Testing Materials

**Figure 3:**
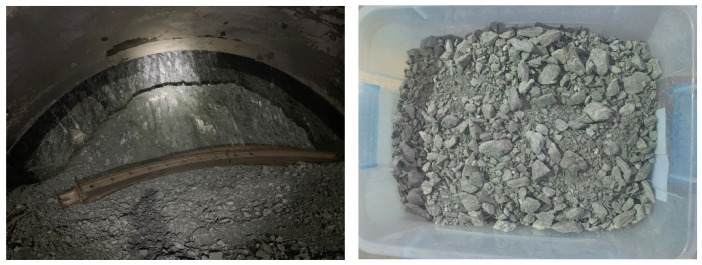
The tunneling site and the collected talus-like rock mass.

The talus-like rock mass used in this test was collected from the Tayi Tunnel (left inset in Figure 3) of Jian-Ge-Yuan Highway Project in Yunnan province in southwestern China [3]. The testing material was collected from the excavation face (right inset in Figure 3) and then transported to the laboratory in moisture-proof storages to retain the natural water content.

The talus-like rock mass experienced a severe weathering effect, leading to a very loose structure and a lower water content of 1.51%. Generally, for the materials in the shearing test, the maximum allowable particle size should be limited according to the size of the shear box [34]. In this study, a maximum rock size of 31.5 mm was determined which is about 1/6.35 of the shear box height, in line with the criterion proposed by Lee et al. [34]. In addition, such a maximum rock size is smaller than 1/5 of the minimum scale of the shear box, ensuring the dimension of the specimen is greater than five times particle size, i.e., a recommended representative elementary volume in laboratory tests [35].

**Figure 4:**
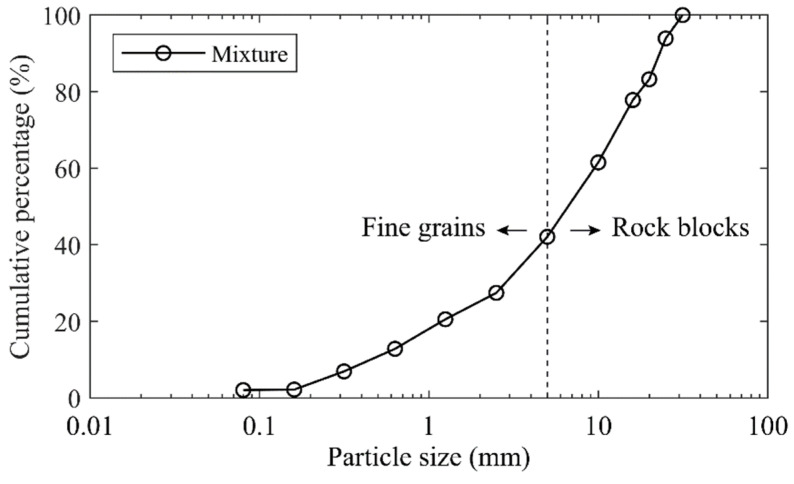
Particle size distribution curve of the mixture of talus-like rock mass.

Generally, the particle size of 5 mm was selected as the threshold separating the soil particles and rock blocks in the soil–rock mixture [13,15,19,22]. This study followed up this criterion, i.e., the particles larger than 5 mm were considered as the rocky blocks, while the ones smaller than 5 mm were treated as the fine grains. Using the standard vibrating screen machine (Figure 2), we obtained the particle size distribution curve (Figure 4) for the original mixture of the talus-like rock mass (i.e., mixture sample) with the following steps: (1) divide the materials into two groups (fine grains and rocky blocks) with a separation of 5 mm with a 5-mm sieve mesh; (2) sieve the fine grains using sieve meshes with mesh size smaller than 5 mm to further obtain the subgroups with different fractions; (3) use sieve meshes > 5 mm to divided the rocky blocks into subgroups; and (4) measure the weight of each subgroup to plot Figure 4. When sieving each group, the sieving time lasted for about 10 mins. On this basis, the block content (weight proportion of rock blocks to the total weight) [17,18,19,22] of the talus-like rock mass was about 57.9%.

**Figure 5:**
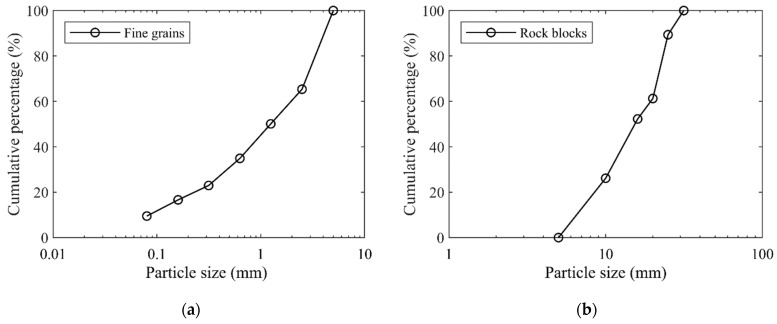
Particle size distribution curves of (**a**) fine grains and (**b**) rocky blocks.

**Figure 6:**
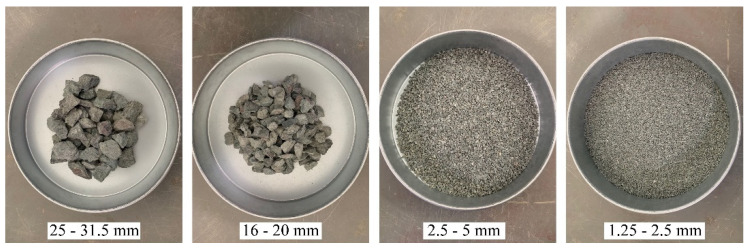
Sieved components with different gradings from natural mixture of the talus-like rock mass.

As stated above, the mechanical properties of pure fine grains (block content = 0%) or rock blocks (block content = 100%) are rarely studied [11]. The component and structure of the talus-like rock mass were extremely complex, represented by the varying-size aggregates formed by fine soil particles, coarse grains and rock blocks. Therefore, both the fabric of mixture mass and the individual fine grains or rocky blocks should be investigated. Beyond the shearing tests on the mixture of talus-like rock mass, this paper also performed shearing tests on the individual fine grains (fine grain sample) and block aggregates (rocky block sample), respectively, which could give some insights into different types of talus-like rock mass and the interaction between them. The particle size distribution curves of the fine grains and rocky blocks are shown in Figure 5a,b, respectively. Figure 6 gives some typical components sieved from the natural mixture.

### 2.3. Testing Procedures

**Figure 7:**
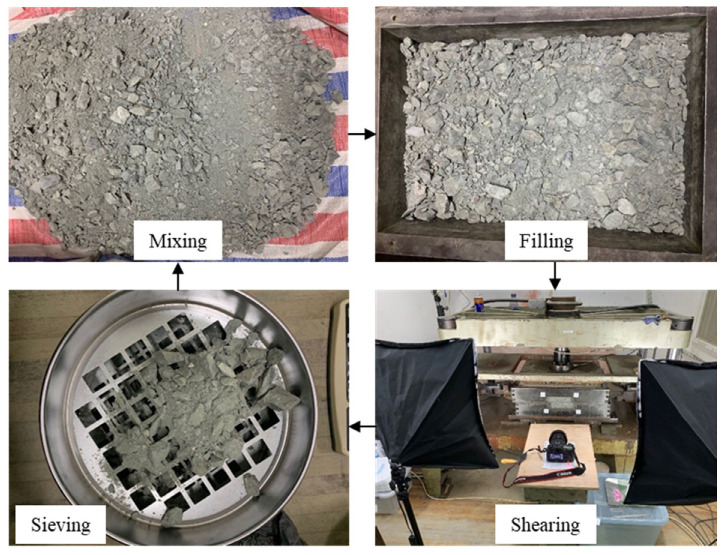
The testing procedures.

The main testing procedures are shown in Figure 7. Before testing, we weighed the components of each grading sieved from natural mixture with the standard vibrating screen machine (Figure 2) according to the measured particle size distribution curve (Figure 4), ensuring that each component of the testing material had the same weight as that of the natural mixture. Each of the measured fractions was then mixed sufficiently by hand to avoid block breakage and to form a shear sample with the same block content as the natural mixture. Afterwards, they were filled into the shear box with three layers. After filling each layer, the specimen was compacted with a soft tamping rammer to densify the specimen. It should be noted that the rammer must be soft enough to avoid breaking the rock blocks. Once the specimen was prepared, a normal stress was specified and then sheared horizontally for about 70 cm shear displacement under a loading speed of 1.6 mm/min. A series of different normal stresses, i.e., 50 kPa, 150 kPa, 250 kPa and 350 kPa, were employed in the tests for the mixture sample, while another series of 50 kPa, 100 kPa, 150 kPa and 200 kPa for the fine grain and rocky block samples were used. After the shearing test finished every time, the specimen was sieved once again to obtain a new grading curve. The difference between the grading curves before and after shearing could provide a basis for analyzing the particle crushing characteristics of the tested material.

### 2.4. Results

The testing apparatus recorded the shear stress of the specimen as a function of the shear displacement of the lower box in the cases of different normal stresses. The strength parameters, i.e., cohesion and friction angle, can be derived from the shear–displacement curves and normal stresses. The dilatancy of the specimen during the shearing process was analyzed by monitoring the normal displacement of the vertical loading plate. In addition, the particle crushing characteristics was analyzed by comparing the grading curves before and after shearing. These testing results were analyzed and presented in the following subsections.

#### 2.4.1. Shear–Displacement Relationship

**Figure 8:**
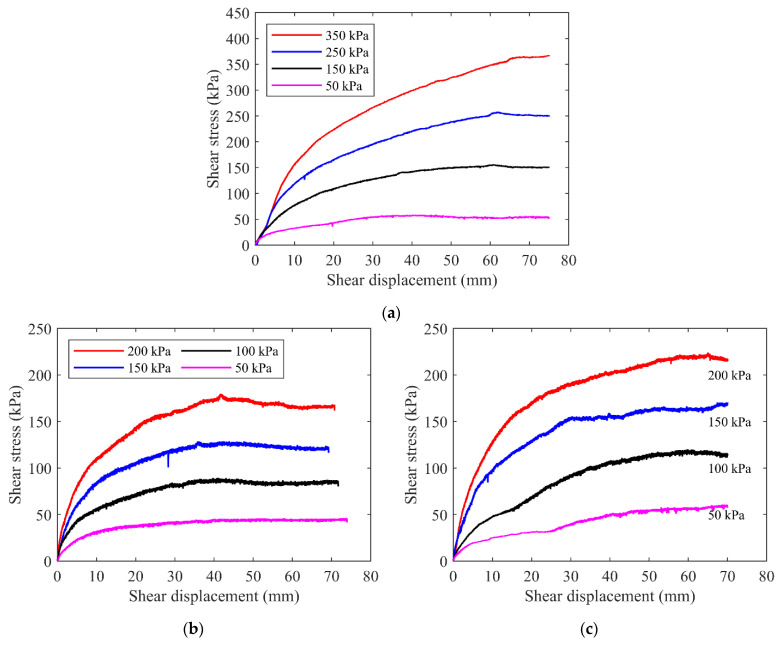
The shear–displacement relationships for (**a**) the mixture of talus-like rock mass, (**b**) fine grains and (**c**) rocky blocks.

The shear–displacement relationships for the three samples of talus-like rock mass under different normal stresses are shown in Figure 8. For the natural mixture sample (Figure 8a), the shear–displacement curves can be characterized as nonlinear, inelastic and stress-dependent. Furthermore, the shear–displacement curves were comprised of three sections: specimen densification, shear damage and plastic flow. Firstly, the curves were nearly linear within a very short stage at the beginning of the test, especially for larger normal stresses. At this stage, the shear stress in the specimen was far smaller than the shearing strength. The specimen deformation was mainly caused by the material densification due to the compaction of the internal voids and the move or flow of the fine grains, thus leading to a linear curve. Afterwards, as the shear displacement increased, the rock blocks translated or rotated more obviously, leading to a closer and more compact contact with each other, which may increase the resistance within the specimen. As a result, the shear stress increased gradually. The closer contact generated the squeezing and rubbing of the rock blocks against each other, which may disturb or even destroy the rock blocks, leading to the particle crushing. Simultaneously, the increase rate of the shear stress reduced gradually. Lastly, the shear stress reached a steady value even though the shear displacement continued to increase. In this stage, a strain hardening phenomenon was observed, which can be also regarded as a plastic flow stage. Moreover, the larger the normal stress is, the later the plastic flow occurs.

The obtained shear–displacement relationships for the mixture of talus-like rock mass showed some similar characteristics presented by previous studies on the soil–rock mixture of similar geomaterials. However, there are still differences between the present talus-like rock mass and the traditional soil–rock mixture [36]. Previous studies have obtained similar plastic stage during the direct shear test, such as Liu et al. [36] and Zhang et al. [18] for soil–rock mixture, Lee et al. [34] for crushed rocks, and Chang and Phantachang [11] for gravel soils. However, the yield stress in their studies emerged at the position about 1/4 [18], 1/3 [36] and 1/2 [11,34] of the total shear displacement, which were earlier than that observed in this study as shown in Figure 8a, especially for higher normal stresses. The later occurrence of yield stress indicated that the mixture of the talus-like rock mass experienced a longer period of stress growth under the action of external force than the traditional soil–rock mixture.

Figure 8b,c shows the shear–displacement relationships for the fine grains and rocky blocks of the talus-like rock mass, respectively, which were also nonlinear, inelastic and stress-dependent. Similarly, three sections for the curves can be recognized, but differences still emerged at the later part of the curves. For the fine grains, when the normal stress was small (e.g., 50 kPa), the plastic flow and strain hardening were observed at the end. However, for larger normal stresses, the weak strain softening phenomenon was observed at the rear part, which became more significant with the increase in the normal stress. For the rocky blocks, the strain hardening appeared at the later part of the curves. The curves of the fine grains were more regular and smoother than those of the rocky blocks. This may be induced by the frequent rolling and interlocking between blocks in the aggregates, resulting in the fluctuation of curves and enlarging of the peak shear stress.

#### 2.4.2. Shear Strength Parameters

**Figure 9:**
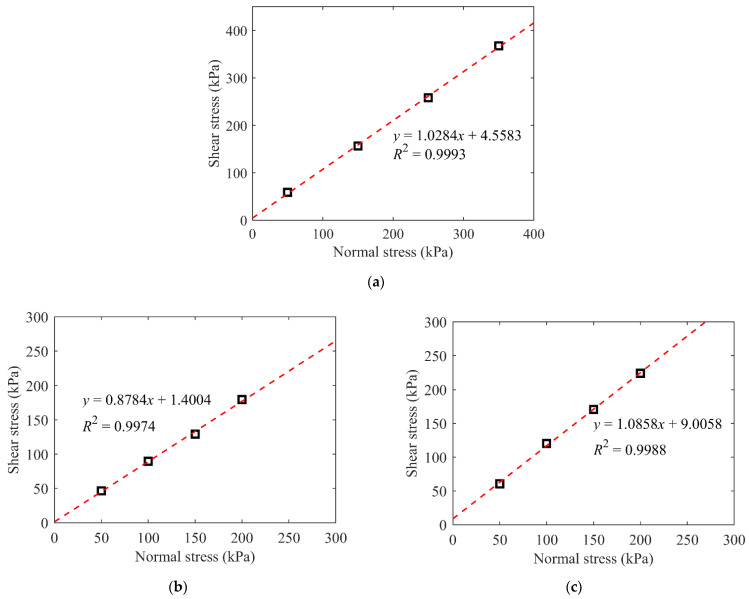
The strength envelopes for (**a**) the natural mixture of talus-like rock mass, (**b**) fine grains and (**c**) rocky blocks.

According to the shear–displacement relationships shown in Figure 8, the strength envelopes for different samples are summarized in Figure 9. The correlation coefficient in each case was greater than 0.99, showing that the normal and shear stresses have a linear relationship. Therefore, the mechanical properties of the talus-like rock mass follow the Mohr–Coulomb criterion, i.e., *τ* = *c* + *σ*·tan*φ*, where *τ*, *σ*, *c* and *φ* are the shear stress, normal stress, cohesion, and friction angle, respectively. The calculated shear strength parameters (cohesion and friction angle) are listed in Table 1. It can be observed that both the cohesion and friction angle increased with the increase in block content. This relationship seems to be different to the previous studies on the soil–rock mixture, where the friction angle increased while the cohesion decreased with the increase in the block content [14,16,22]. The reason may be that, in the studies for the general soil–rock mixture, the soils or fine grains were always the clayey soil, and the internal cohesion between the particles was relatively large. When the rock blocks were mixed into the soil, the soil–rock mixture behaved differently from the pure soil sample, where the soil–rock contacts may reduce the cohesion induced by contacts between soil particles. Nevertheless, the fine grains of the talus-like rock mass used in this study mainly came from the powder-scale particles from the extremely fractured rock mass, which behaved in the form of the sandy soil. In addition, the original water content of the talus-like rock mass in nature was extremely low, i.e., 1.51%, resulting in a weak cohesion. When rock blocks emerged in these kinds of fine grains, an internal skeleton structure was formed and the rock blocks became more dominant in the shearing test as the block content increased. During the shearing process, rock blocks translated and rotated, inducing more contacts and interlockings between themselves. Consequently, the resistance of the rock mass was promoted, and the cohesion and friction angle were both enhanced. When the specimen was fully comprised of rock blocks, the contacts between rock blocks and interlocking effect were more obvious and larger shear strength parameters were obtained. Thus, the individual fine grains or rock blocks both affect the mechanical properties of the talus-like rock mass.

The shear strength parameters in Table 1 also demonstrate that the cohesion was relatively small, i.e., the talus-like rock mass was a typical loose geomaterial with weak internal bonding. This was consistent with the occasional collapse of the Tayi Tunnel during construction [3]. The friction angles between 40° and 50° obtained from shearing tests agreed well with the repose angle of the tunnel collapse deposit in the Tayi Tunnel [3]. In general, the experimental results of the shear strength parameters showed good correlation with the observation in the tunnel site where the specimen was collected, which were useful for the interpretation and guidance of the practical tunneling situation on site.

#### 2.4.3. Dilatancy

**Figure 10:**
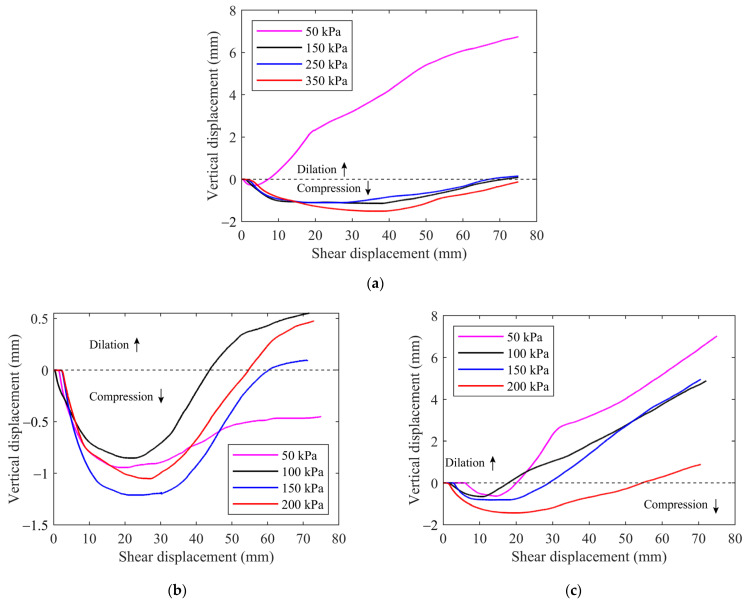
The vertical shear–displacement curves for (**a**) the mixture of talus-like rock mass, (**b**) fine grains and (**c**) rocky blocks.

**Figure 11:**
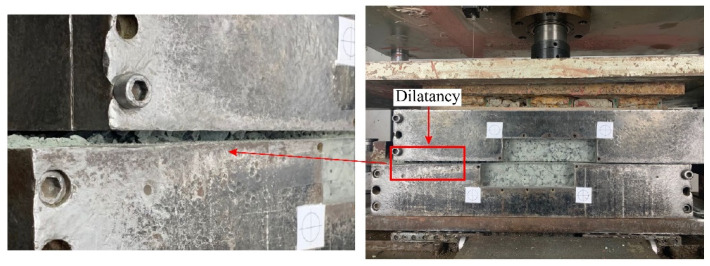
The dilatancy of the talus-like rock mass in the case of 50 kPa normal stress.

The different types of geomaterials may exhibit different deformation characteristics during shearing tests, either dilatancy or compression. The dilatancy feature has a significant effect on the physical and mechanical properties. Figure 10 displays the vertical displacement–shear curves for the three samples at different normal stresses. For the mixture of talus-like rock mass (Figure 10a), the dilatancy was obvious at a low normal stress of 50 kPa, as shown in Figure 11. The dilation of vertical movement may be induced by the translation and rotation of rock blocks as well as climbing over adjacent rock blocks [15], resulting in the volume expansion. A low normal stress of 50 kPa could not fully eliminate the volume expansion. As the normal stress increased, the specimen became more compressive with a large range of vertical displacement observed. The transition from compression to dilation appeared later as the vertical displacement gradual decreased, but the specimen was almost wholly in compression at high normal stress of 350 kPa. The compression happened during the shearing test at higher normal stress, indicating that the mixture of talus-like rock mass has a loose mixed fabric structure with abundant pores inside, which can be easily compressed under large normal stress. The largest normal stress of 350 kPa induced the maximal compression, while the compressive behavior of 150 kPa was similar and even larger than that of 250 kPa. This may be the result of the local inhomogeneity of the internal structure in preparing the specimen and the randomly moved particles during the shearing process.

In the case of pure fine grains, the specimens behaved similarly under different normal stresses (Figure 10b): the vertical displacement first dropped sharply and then gradually turned to climb. When the normal stress was low, i.e., 50 kPa, the fine grains were fully in a state of compression during the shearing test. However, as the normal stress increased, there was a transition from compression to dilation. The reason may be that the installed specimen had a relatively large porosity before testing and the material preserved the loose structure under a very low stress. The internal pores were not fully eliminated as shearing displacement increased, leading to a macroscopic compression. For higher stress, the specimen was pressed and compacted by the loading plate before shearing. As the shear process continued, the dense material was first compressed and then became expansive due to the particles’ translation, rotation or climbing over each other. The material dilation was observed once the expansion exceeded the compression when the shear displacement was over a threshold. In general, a larger normal stress induced a larger compression. However, Figure 10b showed that the normal stresses 100 kPa and 150 kPa induced the minimum and maximum compression, respectively, while the case of 200 kPa had a middle value. In the case of 100 kPa, particles’ dislocation and rotation may generate more voids inside the specimen and enlarge the volume, leading to the minimum compression. When the normal stress reached about 150 kPa, it was difficult for particles to climb over each other and the movement-induced voids were soon supplemented by moving particles. As the normal stress increased to 200 kPa, the high-level stresses may crush large particles whose movement was restricted into smaller ones that could readily move away. Therefore, the compression induced by 200 kPa was smaller than that at 150 kPa. This phenomenon also showed that the dilatancy of the fine grains sieved from naturally talus-like rock mass behaved differently under different stress levels.

As shown in Figure 10c, there was a transmission from compression to dilation for all the four normal stresses. For stresses ranging from 100 kPa to 200 kPa, a larger normal stress induced a larger compression and later emergence of dilatancy. Such a phenomenon was dependent on the evolution of the backbone formed by rock blocks. At the beginning of the test, rock blocks were crushed into small chips that further filled into the internal void of the specimen. The filling effect exceeded the influence by blocks’ movement and rotation, leading to an apparent compression. As the shear displacement increased, rock blocks were redistributed due to increasing shear stress and the specimen became denser. As a result, the volume expansion due to block movement and rotation turned to surpass the compression. The specimen dilatancy was finally observed. For larger normal stress, the compression was more prominent to restrict the volume expansion, leading to a later emergency of dilatancy.

Comparing the dilatancy of the three samples of talus-like rock mass with different block contents, it can be found that the compression mainly happened to the mixture and fine grains because the smaller amount of rock blocks may induce decreased volume expansion. The rocky blocks showed more obvious dilatancy due to the dense contact and adjustment of rock blocks during the shearing test.

#### 2.4.4. Particle Crushing

**Figure 12:**
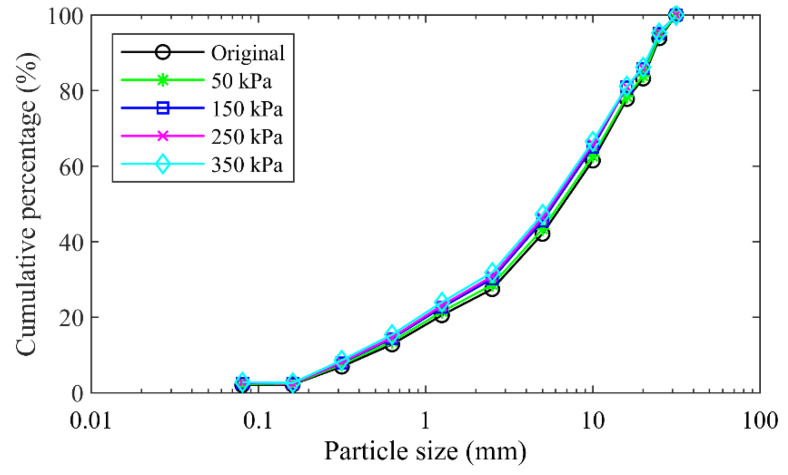
Particle size distribution curves before and after tests for the mixture of talus-like rock mass.

The particle crushing appeared in the specimen formed by the mixture of the talus-like rock mass during the shearing test because of the interaction of rock blocks, which can be demonstrated by the difference between the particle size distribution curves before and after the shearing test, as shown in Figure 12. It was observed that the particle size distribution curves moved upwards after the shearing test under each normal stress, indicating that the size distribution changed after testing due to particle fragmentation. A larger normal stress induced an upper location of the varied particle size distribution curve, i.e., the extent of particle crushing was more obvious in the case of larger normal stress.

**Figure 13:**
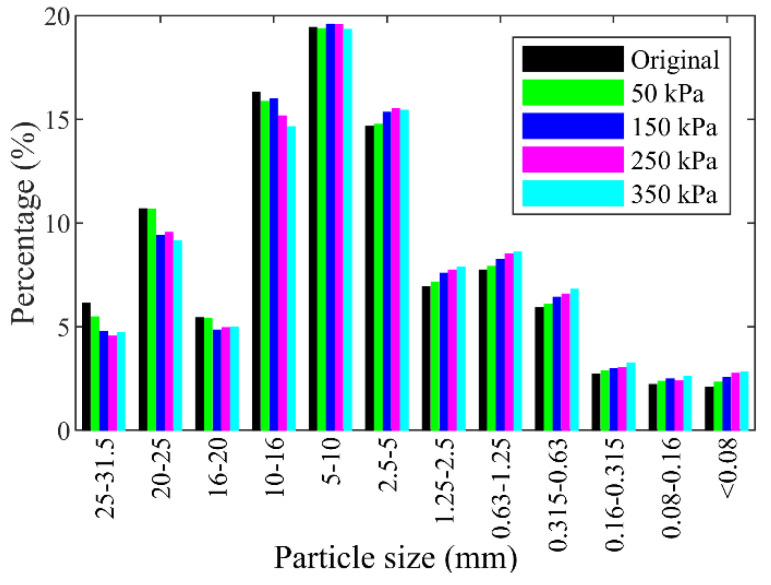
Histogram of contents of each grain size group before and after tests for the mixture of talus-like rock mass.

**Figure 14:**
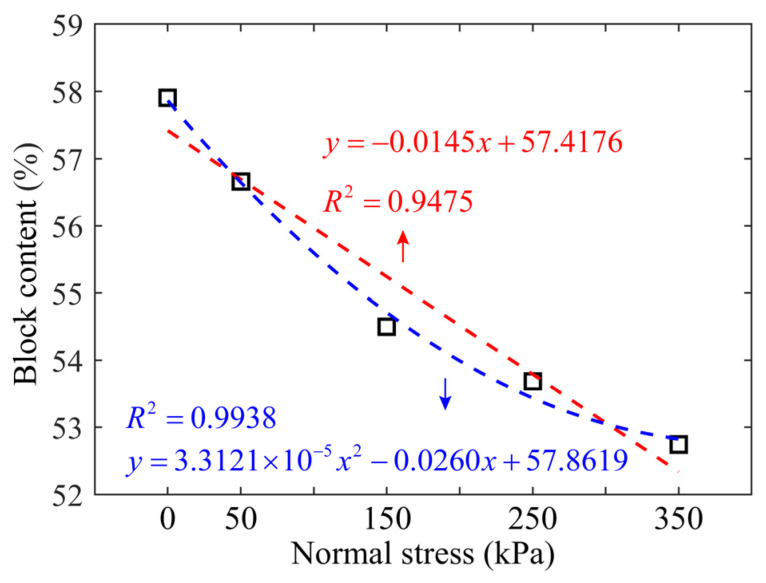
Block content of the mixture sample after the shearing test as a function of normal stress. The value at a zero normal stress indicates the original block content of the mixture, i.e., 57.9%.

Furthermore, the percentages for different individual grain sizes before and after the shearing test are shown in Figure 13. It can be observed that the percentages of particle sizes larger than 10 mm decreased after the shearing test under each normal stress, while that of particle sizes smaller than 5 mm increased. The percentage of the particles in the size range of 5–10 mm remained almost unchanged. The reason may be that the crushing mainly happened to the particles larger than 10 mm, which were crushed into chips smaller than 10 mm or even 5 mm. The particles in the size range of 5–10 mm were crushed into smaller pieces, which may be replenished by the crushing of other particles larger than 10 mm. The proportion of the coarse particles (larger than 5 mm), i.e., the block content, decreased with increasing normal stresses due to the particle crushing, which can be better represented by the quadratic polynomial fitting than a linear function as shown in Figure 14.

**Figure 15:**
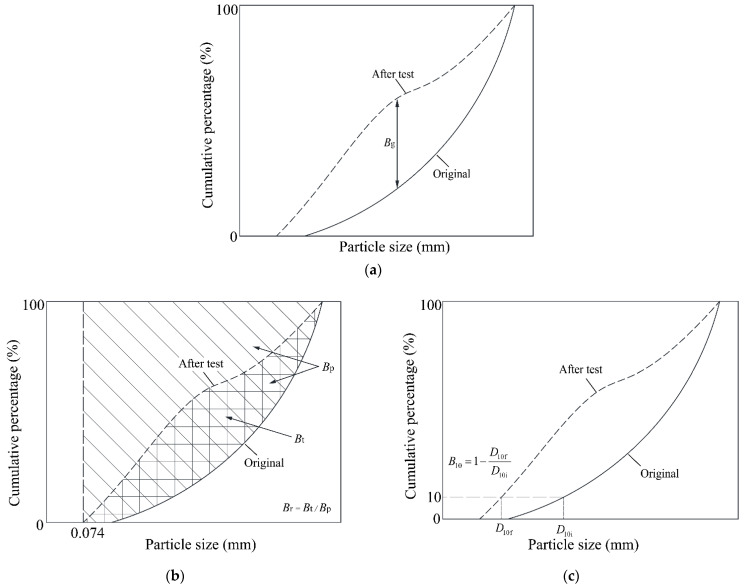
Schematics of particle breakage indices by (**a**) Marsal’s method, (**b**) Hardin’s method and (**c**) Lade’s method.

**Figure 16:**
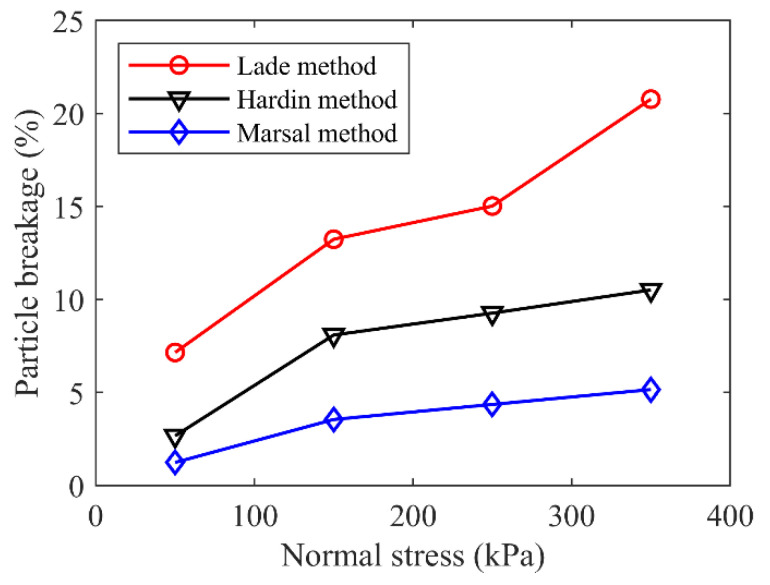
Particle breakage at different normal stresses for the mixture of talus-like rock mass.

The particle crushing characteristics could be further quantified and estimated by employing breakage indices. Three breakage indices, i.e., Marsal’s method [37], Hardin’s method [38] and Lade’s method [39], were employed in this study. Marsal’s breakage index *B*_g_ [37] corresponds to the largest variation in the particle size distribution curves before and after tests (Figure 15a). Hardin [38] defined the breakage potential *B*_p_ as the area between the line of *D* = 0.074 mm and the part of the particle size distribution curve before testing with *D* > 0.074 mm. The total breakage *B*_t_ is defined as the area between the particle size distribution curves before and after testing. Thus, Hardin’s index is derived as *B*_r_ = *B*_t_/*B*_p_ (Figure 15b). Lade [39] noted that *D*_10_, the characteristic particle size with a weight percentage of 10%, is very important, and thus defined a relative breakage *B*_10_ = 1 − *D*_10f/_*D*_10i_ (Figure 15c), where *D*_10i_ is the particle size at a weight percentage of 10% of the original particle size distribution curve and *D*_10f_ is the value after testing. The particle crushing characteristics of the mixture specimen represented by the three indices are displayed in Figure 16. It was clear that all the three indices indicated that a higher normal stress resulted in a larger particle breakage content after the shearing test.

**Figure 17:**
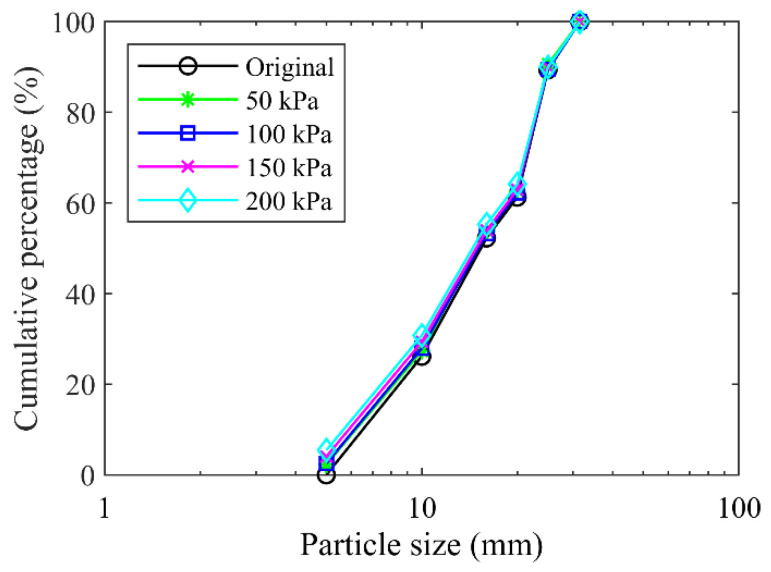
Particle size distribution curves before and after tests for rocky block samples.

**Figure 18:**
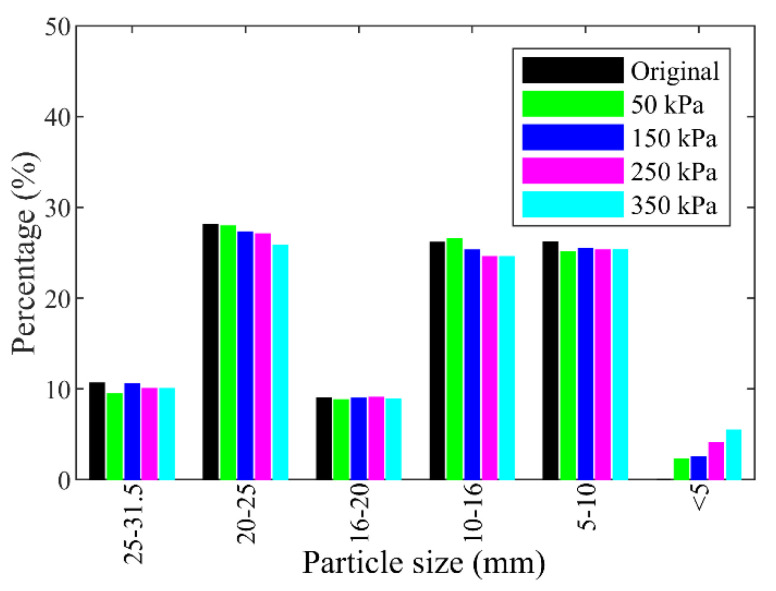
Histogram of contents of each grain size group before and after tests for rocky block samples.

As for the case of rocky blocks, the particle size distribution curves before and after the shearing tests are shown in Figure 17. Similar to Figure 12, the particle size distribution curves moved up after shearing test, and the normal stress had a significant influence on the particle breakage. The percentages for different individual grain sizes before and after the shearing test for the rocky block sample are shown in Figure 18. It was found that all the content of rock blocks decreased after the shearing test except that in the size range of 16–20 mm, which almost remained invariable. The reason is similar to that in the size range of 5–10 mm for the mixture of talus-like rock mass. The fine grains group (smaller than 5 mm) emerged for the crushing of the rock blocks and the content increased as the normal stress increased. The particle crushing characteristics of the rock blocks were similar to that of the mixture of the talus-like rock mass, and it can be inferred that similar quantified breakage indices could be obtain, as in Figure 16.

## 3. DEM Simulation on Shear Test

The heterogeneity and discontinuity of the talus-like rock mass made it difficult to simulate by the traditional continuum mechanics method. In contrast, the DEM-based numerical simulation software Particle Flow Code (PFC) is usually used to study the heterogeneous and discontinuous geomaterial, such as the soil–rock mixture [28,30,31,32,33]. The PFC could simulate the granular material such as soil or sand by a series of discrete disks (PFC2D) or balls (PFC3D) based on the interaction between them according to Newton’s theorem of motion. Recently, some more built-in models such as clump [28,33] and cluster (parallel bond model) [13,31,32,40] have been developed to model the rigid rock blocks by bonding the disks or balls together. Thus, it is also possible to simulate the different types of talus-like rock mass composed of both fine grains and rock blocks. In this section, the PFC2D was used to carry out the DEM simulations on shear test for the three types of talus-like rock mass mentioned above.

### 3.1. Model Setup

**Figure 19:**
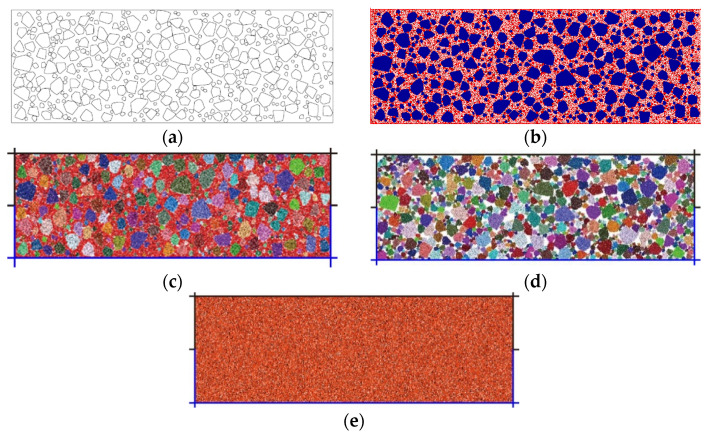
The generation of rock blocks in the talus-like rock mass: (**a**) generation of convex polygons; (**b**) blocks and fine grains; (**c**) model of mixture; (**d**) model of rocky blocks; (**e**) model of fine grains.

Based on the random extension algorithm for polygonal block generation by Xu et al. [24], this paper generated a series of nonoverlapping convex polygons to model the rock blocks according to the size distribution curves, as shown in Figure 19a. The polygons were then converted to rigid blocks (blue polygons in Figure 19b) and disks with radii ranging between 0.6 to 0.9 mm were generated to fill up these polygons. Among the blocks, disks were generated to model the fine grains (red particles in Figure 19b). After assigning different micro parameters to rigid blocks and fine grains, the final model of the mixture of talus-like rock mass is shown in Figure 19c. The model of rocky blocks (Figure 19d) was established by using similar method without considering the generation of disks outside the polygons. Since there may be a large number of disks and the calculation efficiency of the DEM simulation would be reduced if all the real sizes of fine grains were considered [24], this paper only considered two groups of fine grains from 1.25 to 5 mm. The fine grains smaller than 1.25 mm were replaced by that in the range of 1.25 to 2.5 mm. The numerical model for the pure fine grains specimen was established similarly (Figure 19e). This would be feasible if the mechanical behavior agreed with that of the experimental results [24]. In this study, the rolling resistance model [32,41] was employed to represent the contacts between the fine grains and rock blocks. Because the rock blocks could be crushed during the shearing test, the linear parallel bonding model was established to simulate this behavior by bonding the disks into the blocks together [13,31,32,40].

### 3.2. Calibration of Micro Parameters

**Figure 20:**
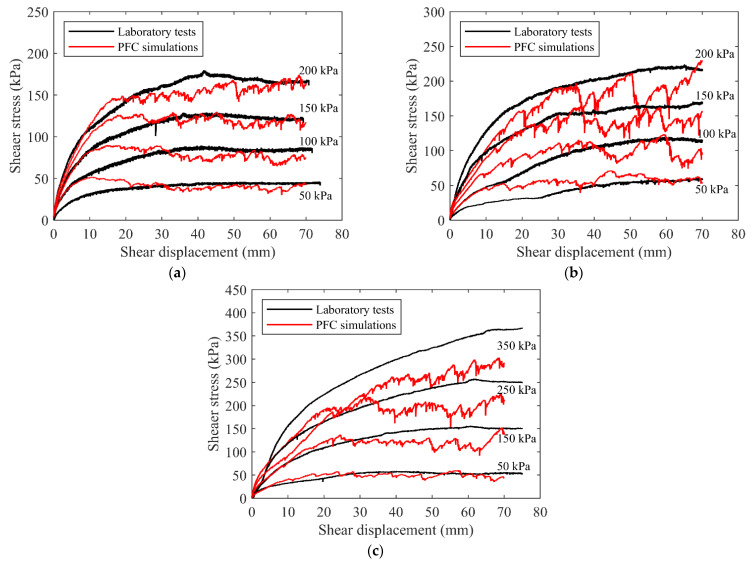
The shear–displacement relationships from the numerical and laboratory tests for (**a**) fine grains, (**b**) rocky blocks and (**c**) the mixture of talus-like rock mass.

Due to the lack of the relationship between the macro parameters of the talus-like rock mass and the micro parameters of the particles and contacts, we used the trial-and-error method to calibrate the micro parameters for DEM simulations. The calibration procedures mainly consisted of four steps: (1) specify an initial value for each parameter according to previous studies given the particle size and execute the calibration model (Figure 19c–e) to obtain the shear stress–displacement relationship; (2) change the elastic modulus and the stiffness ratio of normal to tangential contact to achieve a relative similar curve to the laboratory tests; (3) given the above two parameters, change other parameters to obtain a more suitable curve to the laboratory one; and (4) rerun Steps (2) to (3) to finally obtain a consistent shear stress–displacement relationship with the laboratory shear test. The calibration models were bounded by rigid walls forming upper and lower shear boxes, represented by the black and blue lines (Figure 19c–e), respectively. As shown in Figure 20a, the shear stress–displacement relationships of the fine grains obtained from both the numerical and laboratory tests were very similar to each other under the same normal stress. The numerical results of the rocky blocks showed obvious fluctuation in Figure 20b. This may be attributed to the reduced degrees of freedom of rock blocks in the 2D simulation model, where the motion of rock blocks was limited to a 2D plane. The interaction between the rock blocks and that between the rock blocks and shear box may lead to the fluctuation of the curves. However, the final results of the numerical still agreed well with the curves of the laboratory tests. The calibrated parameters for both fine grains and rocky blocks were listed in Table 2.

After the calibration of parameters for both fine grains and rocky blocks, the obtained parameters were used for modeling the mixture of talus-like rock mass. The lithology of the fine grains may be consistent with that of the blocks for that the fine grains mainly came from the rock debris or the powders by the breakage of rock blocks. Therefore, the contact parameters of the fine grain–rock block inherited that from the rock–rock contacts for the sake of simplicity, as listed in Table 2. For this aspect, Han et al. [32] also showed that the micro parameters for the soil–rock contact and block–block contact were very similar to each other for the DEM model of the soft soil–rock mixtures. The numerical results of the mixture of talus-like rock mass against the tests are shown in Figure 20c. It can be noted that, in the cases of normal stresses of 50 kPa and 150 kPa, the numerical results agreed well with the experimental ones. However, there was an obvious difference between them in the cases of larger stresses. It may be explained that the micro parameters for both fine grains and rocky blocks were calibrated under a maximum normal stress of 200 kPa, which was smaller than that in the test of mixture. Thus, the numerical results showed certain deviation under the conditions where the normal stress surpassed 200 kPa. On the other hand, the talus-like rock mass was more frequently encountered on the surface or in shallow formations, which may not suffer such a large normal stress. Therefore, we believe that the calibrated micro parameters are able to reflect the mechanical behavior of the talus-like rock mass.

### 3.3. Simulation Results

The DEM simulation could reveal internal microscopic phenomena, such as the force chains and bond-break evolution, which could not be captured during the laboratory test. In addition, we compared the dilatancy via DEM model and made a comparison with the laboratory tests.

#### 3.3.1. Force Chain

**Figure 21:**
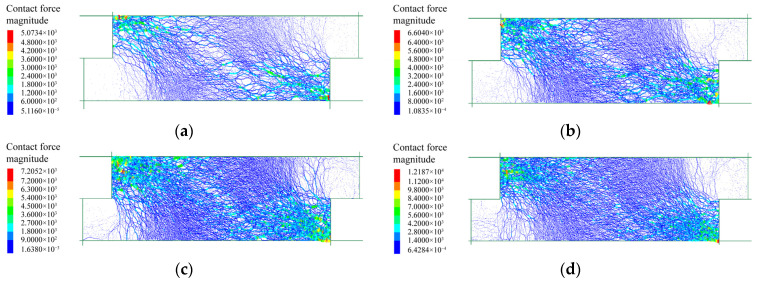
The force chain distributions of fine grains after the shearing test under different normal stresses: (**a**) 50 kPa; (**b**) 100 kPa; (**c**) 150 kPa; (**d**) 200 kPa.

The internal force chain of the specimen was generated by assembling the contact forces between disks, which reflected the true stressed region during shearing tests. The internal force chain distributions of fine grains under different normal stresses are shown in Figure 21. The force chain mainly developed in the region from top left to bottom right. Only a few force chains were generated in the regions of the top right and bottom left. The force chains became denser as the normal stress increased, meaning that the internal force increased and more particles were mobilized to sustain the shearing action under higher normal stress. Furthermore, an oblique sparse band was formed in the middle of the model, demonstrating that a shear slip zone developed accordingly. It can also be seen that the shear slip zone became narrow under higher normal stress.

**Figure 22:**
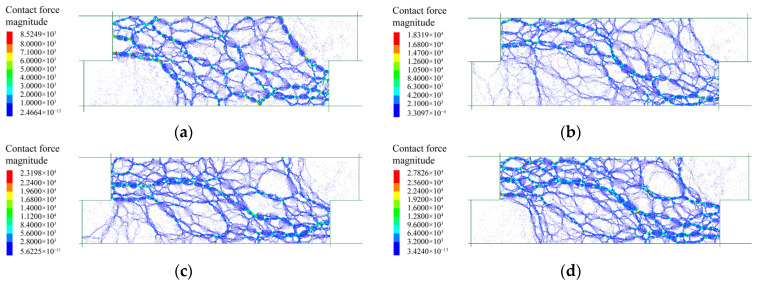
The force chain distributions of rocky blocks after the shearing test under different normal stresses: (**a**) 50 kPa; (**b**) 100 kPa; (**c**) 150 kPa; (**d**) 200 kPa.

Figure 22 shows the force chain distribution of rocky blocks under different normal stresses. A distribution region from top left to bottom right, similar to that of the fine grains, was observed, and higher normal stress promoted denser and stronger force chain. Nevertheless, an obvious difference was observed in that the force chains formed in rocky blocks were generally coarser than those generated by fine grains, indicating that the block–block contacts sustained larger stresses. Such a phenomenon showed that the shear displacement was mainly withstood by the backbone formed by rock blocks. The skeleton became denser under higher normal stress to better sustain the increased stress. For another, the force chain was continuous without the formation of an obvious shear slip zone, meaning that a continuous and stable force skeleton had been generated after the shearing tests.

**Figure 23:**
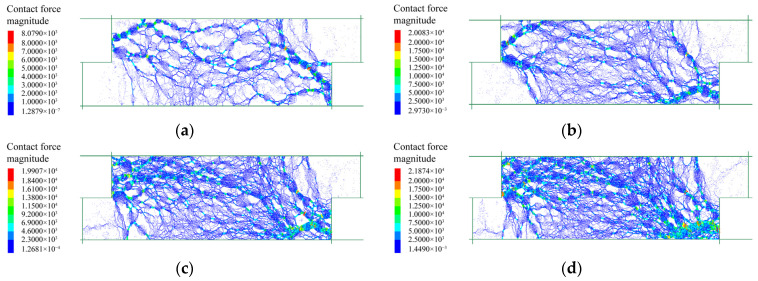
The force chain distributions of the mixture sample after shearing test under different normal stresses: (**a**) 50 kPa; (**b**) 150 kPa; (**c**) 250 kPa; (**d**) 350 kPa.

The force chain distributions of the mixture of talus-like rock mass are shown in Figure 23. For the normal stresses of 50 kPa and 150 kPa, force chains were generated similarly to those in Figure 22, demonstrating that the shearing action was mainly sustained by the rock skeleton inside the mixture. For higher normal stresses, the force chains became denser and an increasing number of thinner force chains were generated, indicating that the fine grains also sustained the shear force as one of the main participants. Another reason may be that the rock blocks could be crushed under higher normal stresses and thus the skeleton effect of rock blocks was weakened.

#### 3.3.2. Coordination Number

**Figure 24:**
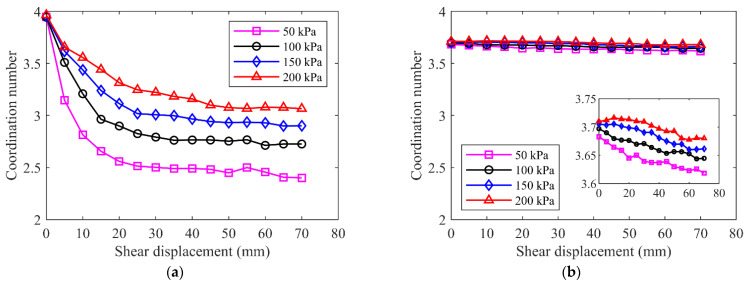

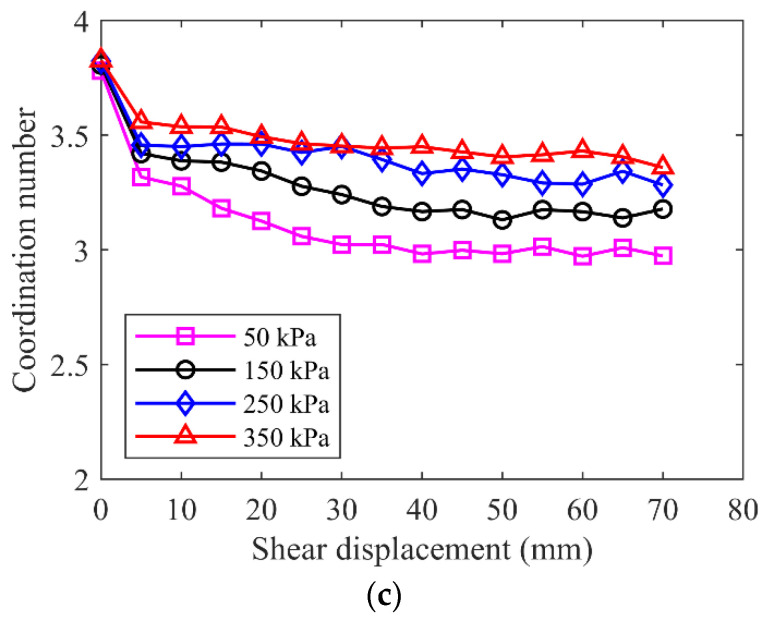
Evolution of coordination number during shearing test: (**a**) fine grains; (**b**) rocky blocks; (**c**) the mixture of talus-like rock mass.

The coordination number is the average number of contacts per particle and a measure of the packing density of a granular assembly, which is defined as *Z* = 2*Nc*/*Np*, where *Nc* and *Np* are the numbers of contacts and particles, respectively [42,43]. Figure 24 showed the evolution of the coordination number during shearing test for the three samples. At the initial state, the specimens were fully consolidated under different normal stresses and the higher normal stress induced a larger coordination number, but the differences were not obvious. The specimen of pure fine grains had the largest coordination number before shearing, meaning a maximum packing density inside. The emergence of rock blocks decreased the coordination number in the mixture of talus-like rock mass. The pure rock blocks had the minimum coordination number because of the skeleton formation and large internal porosity. In general, the coordination number decreased as the shear displacement increased, indicating that the initial fabrics were destroyed during shearing. The higher normal stress promoted a larger coordination number and more compacted fabric. For the fine grains and mixture, the coordination number decreased rapidly at the beginning of shearing due to the sudden change of the state, and gradually reached a steady value, which was similar to the observation by Kodicherla et al. [44]. The coordination number dropped more slowly under higher normal stress, showing a more stable fabric [45]. As for the rocky blocks, there was no obvious differences in the decrease rate of the coordination number, indicating that there was no sudden change for the state and fabric of rock blocks under different normal stresses. This may be attributed to the obvious strong rock skeleton effect, similar to the observation on the railway ballast by Bian et al. [46], which was analogous with the blocks herein. The coordination number of the mixture and rocky block samples fluctuated more violently than the fine grains due to the emergence and interaction of rock blocks, leading to the nonuniform variation of the fabric during shearing. This phenomenon agreed well with the obvious fluctuations of the shear–displacement curves of the rock blocks and mixture in Figure 20b,c. At the end of shearing, the specimen of fine grains had the minimum coordination number and the rocky blocks had the maximum value, which was opposite to the situations at the beginning. This indicated that the fine grains sustained more damages in fabric than the other two specimens, and rocky blocks could help stabilize the fabric during the shearing. As a result, the final value of coordination number in the specimen formed by pure blocks was larger than that in the mixture.

#### 3.3.3. Bond-Break Evolution

The parallel bonding model was employed in this paper to model the rock blocks by bonding the particles belonging to the rock blocks together. During the shear process, the bonds between particles would be broken if the stress exceeded the bonding strength [13]. As a result, a bond break would be created and thus recorded. Therefore, we were able to analyze the particle crushing characteristics in the DEM simulations according to the bond-break evolution.

**Figure 25:**
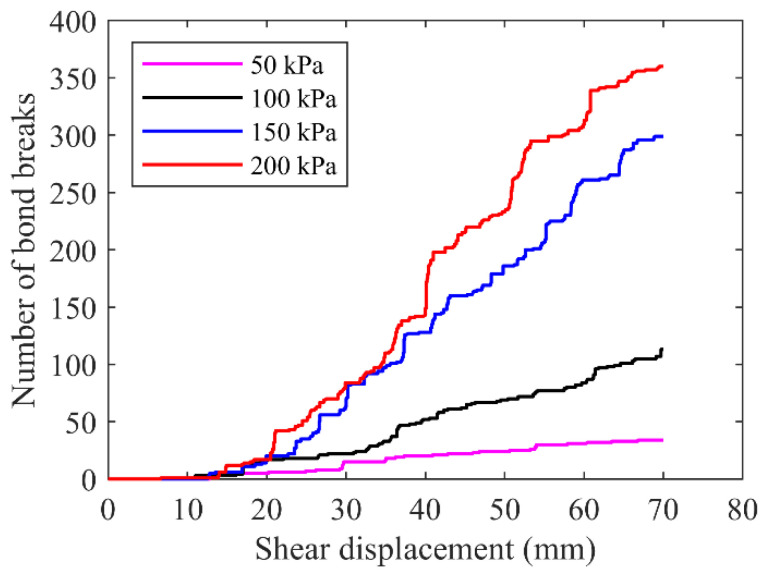
The development of bond breaks for the rocky blocks.

The bond-break development inside the rocky block sample under different normal stresses are summarized in Figure 25. The bond breaks emerged at the shear displacement of about 1–1.5 cm and increased obviously with the increasing shear displacement. A higher normal stress increased the bond-break number and promoted the increase rate. The bond-break numbers after test of normal stresses of 150 kPa and 200 kPa were close to each other and larger than those of the rest two small normal stresses. It could be inferred that the fractures would develop fast if increase the normal stress from 100 kPa to 150 kPa, while the growth rate may be reduced if the normal stress exceeded 150 kPa.

**Figure 26:**
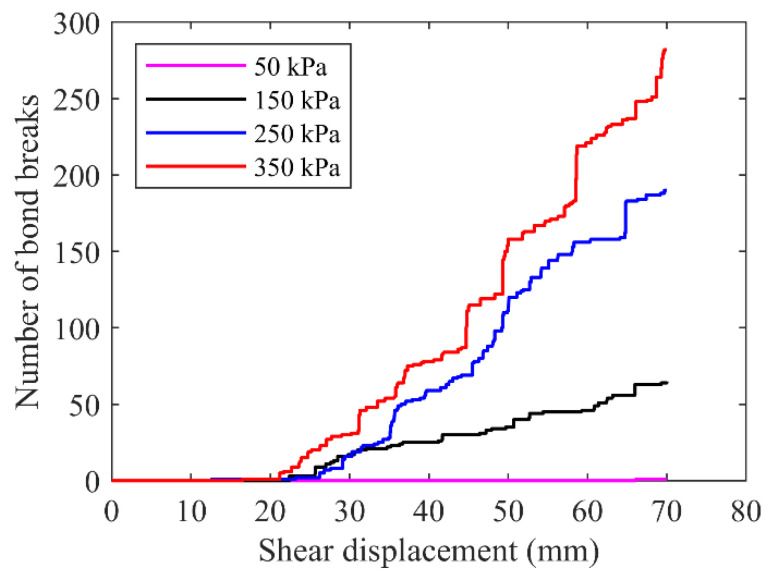
The development of bond breaks for the mixture of talus-like rock mass.

Figure 26 showed the bond-break number as a function of shear displacement for the mixture sample, where similar phenomena to the case of rocky blocks were observed. It was obviously different that the bond-break number under each normal stress was less than that of the specimen formed by pure blocks. The emergency time of the first bond break was later than that in Figure 25. This was due to the reduction in the block content in the mixture and the presence of fine grains reduced the contacts and interaction between rock blocks.

**Figure 27:**
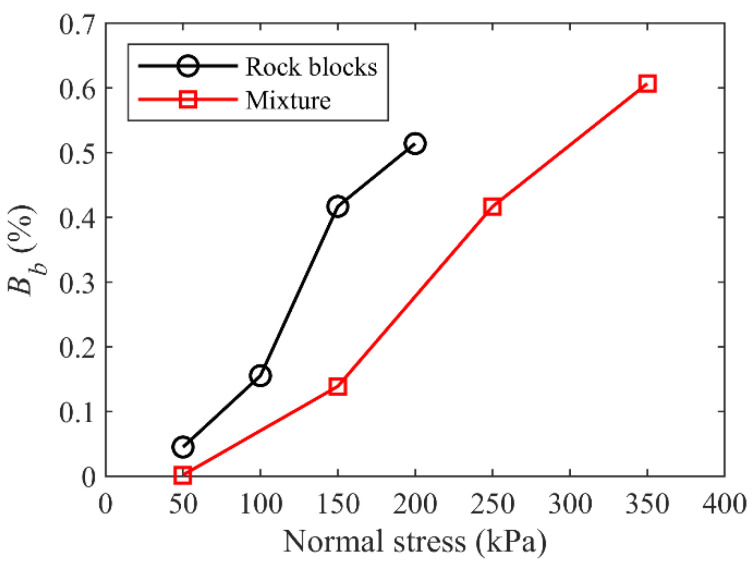
The meso-ratio of block breakage *B_b_* for rocky block and mixture samples.

In order to evaluate the particle crushing characteristics quantitatively, the meso-ratio of block breakage *B_b_* [32] was used as follow:

(1) $$B_{b}=\frac{N_{\mathrm{bond}-\mathrm{break}}}{N_{\mathrm{bond}}}\times 100\%,$$

where *N*_bond-break_ was the total number of broken bonding contacts and *N*_bond_ was the total number of bonding contacts before shearing test. The *B_b_* values for both rocky block and mixture samples of talus-like rock mass are shown in Figure 27. It was found that the proportion of broken bonds had a linear positive relationship with the normal stress, which was consistent with the observations by Zhang et al. [32] and the laboratory tests shown in Figure 16. Moreover, the model of rocky blocks had a larger value of *B_b_* than the mixture model under the same normal stress, same as that the increase in VBP promoted the breakage of blocks [32].

## 4. Conclusions

This paper investigated the shearing characteristics of three samples of talus-like rock mass, i.e., the mixture, fine grain and rocky block samples, based on the laboratory shear tests and DEM numerical simulations. The main conclusions are summarized as follows:

(1) In the laboratory tests, it was observed that the strain hardening phenomenon mainly occurred at the later of the shearing tests for the mixture and rocky block samples. For the fine grains, the small normal stress (e.g., 50 kPa) induced plastic flow and strain hardening whereas larger normal stresses promoted weak strain softening. Both the cohesion and friction angle increased from fine grains to rocky blocks, showing a dependency on the increasing block content. It was also found that the higher normal stress induced less dilatancy and the increased block content promoted the dilatancy.
(2) The laboratory tests showed that the particle crushing phenomenon became more obvious as the normal stress increased, resulting in the decrease in coarse particle proportion and the increase in fine grain proportion. Meanwhile, the particles in the middle range remained almost unchanged for both the mixture and rocky block samples.
(3) In the DEM-based numerical simulations, the dependency of the force chains on the block content was obtained. The fine grain specimen mainly generated uniform thin force chains while coarser force chains were formed in the samples with higher block content, i.e., the rocky block and mixture samples. In addition, the obvious rock skeleton was observed in the models of rocky blocks and the mixture, implying that the coarse grains were the main bearing component as block content increased.
(4) The evolution of the coordination number was also found to be dependent on the block content, showing that the rocky blocks inside the specimen could help stabilize the fabric during shearing, consistent with the gradual decrease in coordination number during shearing. It is also observed that the larger normal stress promoted larger coordination number and more compacted and stable fabric during the shear process.
(5) The bond-break evolution obtained by the DEM simulation revealed its dependency on the normal stress, i.e., higher normal stress increased the bond-break number and promoted the increase rate. Consequently, the meso-ratio of block breakage had a positive relationship with the normal stress. It was also found that more bond breaks occurred inside the rocky block specimen than in the mixture of the talus-like rock mass.

The phenomena captured by the laboratory shear tests and the microscopic observations in the DEM simulations threw light on the shearing characteristics of the talus-like rock mass. The findings will help us to better understand the interaction between the surrounding rock mass and the tunnel support system, and thus be beneficial to optimizing the design of the tunnel supporting and mitigating the engineering disasters during tunnel construction.

## Figures and Tables

**Table 1:** The shear strength parameters for different types of talus-like rock mass.

Types	Block Content (%)	Cohesion (kPa)	Friction Angle (°)
Fine grains	0	1.40	41.3
Mixture	57.9	4.56	45.8
Rock blocks	100	9.01	47.36

**Table 2:** The micro parameters calibrated in the DEM model.

Material	Contact Model	Parameter	Value
Fine grains	Rolling resistance model	Elastic modulus of contacts (MPa)	70.0
		Stiffness ratio of normal to tangential contact	1.5
		Friction coefficient	0.5
		Rolling resistance coefficient	3.0
Rocky blocks	Parallel bonding model	Elastic modulus of contacts (MPa)	100.0
		Stiffness ratio of normal to tangential contact	1.5
		Friction coefficient	0.45
		Elastic modulus of the parallel bond (MPa)	100.0
		Stiffness ratio of normal to tangential of the parallel bond	1.5
		Parallel bond tensile strength (MPa)	8.0
		Parallel bond cohesion (MPa)	4.0
		Parallel bond frictional angle (°)	45.0
Contacts of fine grain–block and block–block	Rolling resistance model	Elastic modulus of contacts (MPa)	100.0
		Stiffness ratio of normal to tangential contact	1.5
		Friction coefficient	0.45
		Rolling resistance coefficient	1.0

## Data Availability

Not applicable.
